# Revised Tool for the Quality Assessment of Diagnostic Accuracy Studies Using AI (QUADAS-AI): Protocol for a Qualitative Study

**DOI:** 10.2196/58202

**Published:** 2024-09-18

**Authors:** Ahmad Guni, Viknesh Sounderajah, Penny Whiting, Patrick Bossuyt, Ara Darzi, Hutan Ashrafian

**Affiliations:** 1 Institute of Global Health Innovation Imperial College London London United Kingdom; 2 Population Health Sciences Bristol Medical School University of Bristol Bristol United Kingdom; 3 Department of Epidemiology & Data Science Amsterdam University Medical Centres Amsterdam Netherlands

**Keywords:** artificial intelligence, AI, AI-specific quality assessment of diagnostic accuracy studies, QUADAS-AI, AI-driven, diagnostics, evidence synthesis, quality assessment, evaluation, diagnostic, accuracy, bias, translation, clinical practice, assessment tool, diagnostic service

## Abstract

**Background:**

Quality assessment of diagnostic accuracy studies (QUADAS), and more recently QUADAS-2, were developed to aid the evaluation of methodological quality within primary diagnostic accuracy studies. However, its current form, QUADAS-2 does not address the unique considerations raised by artificial intelligence (AI)–centered diagnostic systems. The rapid progression of the AI diagnostics field mandates suitable quality assessment tools to determine the risk of bias and applicability, and subsequently evaluate translational potential for clinical practice.

**Objective:**

We aim to develop an AI-specific QUADAS (QUADAS-AI) tool that addresses the specific challenges associated with the appraisal of AI diagnostic accuracy studies. This paper describes the processes and methods that will be used to develop QUADAS-AI.

**Methods:**

The development of QUADAS-AI can be distilled into 3 broad stages. Stage 1—a project organization phase had been undertaken, during which a project team and a steering committee were established. The steering committee consists of a panel of international experts representing diverse stakeholder groups. Following this, the scope of the project was finalized. Stage 2—an item generation process will be completed following (1) a mapping review, (2) a meta-research study, (3) a scoping survey of international experts, and (4) a patient and public involvement and engagement exercise. Candidate items will then be put forward to the international Delphi panel to achieve consensus for inclusion in the revised tool. A modified Delphi consensus methodology involving multiple online rounds and a final consensus meeting will be carried out to refine the tool, following which the initial QUADAS-AI tool will be drafted. A piloting phase will be carried out to identify components that are considered to be either ambiguous or missing. Stage 3—once the steering committee has finalized the QUADAS-AI tool, specific dissemination strategies will be aimed toward academic, policy, regulatory, industry, and public stakeholders, respectively.

**Results:**

As of July 2024, the project organization phase, as well as the mapping review and meta-research study, have been completed. We aim to complete the item generation, including the Delphi consensus, and finalize the tool by the end of 2024. Therefore, QUADAS-AI will be able to provide a consensus-derived platform upon which stakeholders may systematically appraise the methodological quality associated with AI diagnostic accuracy studies by the beginning of 2025.

**Conclusions:**

AI-driven systems comprise an increasingly significant proportion of research in clinical diagnostics. Through this process, QUADAS-AI will aid the evaluation of studies in this domain in order to identify bias and applicability concerns. As such, QUADAS-AI may form a key part of clinical, governmental, and regulatory evaluation frameworks for AI diagnostic systems globally.

**International Registered Report Identifier (IRRID):**

DERR1-10.2196/58202

## Introduction

Despite many promises, the integration of artificial intelligence (AI)–centered systems into clinical workflows has been limited thus far. In the current paradigm, diagnostic investigations require interpretation from expert clinicians in order to generate a diagnosis and subsequently determine management. However, diagnostic services across the world are overburdened with unmanageable workloads, which exceed workforce capacity [1]. In order to address this, diagnostic AI systems have been characterized by regulators and technologists as medical devices [2] that may achieve diagnostic accuracy comparable to that of an expert clinician, while concurrently alleviating health-resource use, helping to reduce medical errors. Indeed, the majority of health care–related AI systems that have reached regulatory approval belong to the field of medical diagnostics [3]. As seminal primary research studies arise on the theme of AI diagnostics [4,5], there has been a concomitant rise in secondary research studies that amalgamate the findings of comparable studies.

Although systematic reviews serve an important role in summarizing evidence, the vast majority related to AI diagnostic accuracy have been conducted in the absence of an AI-specific methodological quality assessment tool [6]. AI diagnostic accuracy studies are methodologically distinct from traditional diagnostic accuracy studies as they comprise distinct methods, analyses, and outcome measures that mandate specific considerations when assessing quality [7]. Currently, the most commonly used instrument for the methodological assessment of secondary research studies remains the quality assessment of diagnostic accuracy studies (QUADAS-2) tool [8]. It is a quality assessment tool designed for use in systematic reviews, initially developed in 2003 [9] and updated in 2011; its use is strongly encouraged by many biomedical journals. It consists of four key domains: (1) patient selection, (2) index test, (3) reference standard, and (4) flow and timing. These domains allow researchers to undertake a structured appraisal of a research study’s internal validity (biases) and external validity (applicability), respectively. The absence of a robust quality assessment tool in the AI field not only hinders efficient quality appraisal at an evidence synthesis phase but has considerable downstream effects as key stakeholders, such as policy makers, regulatory officials, technologists, and health care professionals, are unable to effectively evaluate the translational potential of these nascent technologies.

We propose an AI-specific extension, termed AI-specific QUADAS (QUADAS-AI), that aims to provide researchers and policy makers with a framework to appraise methodological quality in systematic reviews evaluating the diagnostic accuracy of AI. This work is complementary to the Standards for Reporting Diagnostic accuracy studies (STARD-AI) [10] and QUADAS-3 initiatives. QUADAS-AI is being coordinated by a project team and a steering committee consisting of clinician scientists, computer scientists, journal editors, Enhancing the Quality and Transparency of Health Research (EQUATOR) Network representatives, regulatory leaders, epidemiologists, statisticians, industry leaders, funders, health policy makers, legal experts, and bioethicists. Given the global reach of this class of technologies and the transformative potential in clinical diagnostics, we view that connecting global stakeholders is of the utmost importance for this initiative. This study aims to produce a novel quality assessment tool (QUADAS-AI) that accounts for the specific considerations required for the appraisal of AI diagnostic accuracy studies.

## Methods

### Overview

This protocol has benefitted from the experience and expertise of members of the project team and steering committee who have previously led the development of seminal quality assessment tools over the past 2 decades. These include QUADAS and QUADAS-2 for diagnostic accuracy studies, risk of bias in systematic reviews (ROBIS) for systematic reviews [11], and prediction model risk of bias assessment tool (PROBAST) [12] for prediction modeling studies. Moreover, there is shared learning from the development of AI-specific reporting guidelines and risk of bias tools, including STARD-AI [10] and PROBAST-AI [13]. The development of QUADAS-AI can be distilled into 3 broad stages, as previously delineated [14]. Given the pressing need for suitable quality assessment standards of diagnostic studies in this field, development is projected to finish by the end of 2024.

### Stage 1

#### Project Organization

QUADAS-AI is being undertaken by a project team and a steering committee. The project team consists of the founder of QUADAS (PW), the lead for the STARD-AI initiative (HA), and a clinician scientist (AG). The project team is responsible for identifying members of the steering committee, candidate item generation, undertaking the online surveys for the modified Delphi consensus process, organizing the consensus meeting, drafting the QUADAS-AI tool and accompanying documents, coordinating the piloting of the draft QUADAS-AI tool, and leading the dissemination process.

The steering committee was created in order to provide diverse stakeholder guidance in this process, as well as to identify additional experts to invite for the consensus response and draft the final QUADAS-AI tool. The steering committee currently comprises approximately 15 members and consists of health care professionals, computer scientists, epidemiologists, statisticians, regulatory officials, health policy leaders, and industry leaders. These individuals were identified through their notable work in the fields of (1) diagnostic accuracy research, (2) AI in health care, and (3) applied health policy.

#### Defining Scope

The scope of QUADAS-AI has been defined by the project team and steering committee through a discussion framed around questions previously proposed [14]. It was predetermined that QUADAS-AI, as per previous iterations of the tool, would focus on the methodological quality of AI diagnostic accuracy studies. This study is complementary to the ongoing QUADAS-3 initiative, which is the next iteration of QUADAS and is currently led by one of the study authors and the project team (PW). If a draft of the QUADAS-3 becomes available during the development of QUADAS-AI or any substantial updates are anticipated in comparison to QUADAS-2, we will base the QUADAS-AI tool on the QUADAS-3 structure; otherwise, we will instead focus on QUADAS-2. Discourse related to the (1) assessments related to the risk of bias (internal validity), (2) assessments related to applicability (external validity), (3) tool structure, and (4) rating system is a dynamic process that will be open to adaptation throughout stage 2 of the study.

### Stage 2

#### Item Generation

In order to generate a candidate list of items to enter the modified Delphi consensus process, the project team will undertake a mapping review, a meta-research study, a scoping survey with a global panel of experts, and a patient and public involvement and engagement (PPIE) exercise.

#### Mapping Review

A mapping review of both academic and nonacademic literature has been undertaken in order to identify key considerations in the development of QUADAS-AI. An electronic database search of MEDLINE and Embase was conducted through Ovid (Wolters Kluwer). This process was augmented by nonsystematic searches using traditional search engines for gray literature, social networking platforms, as well as personal paper collections highlighted by members of the project team. The extracted material was broadly classified into four categories: (1) general considerations regarding diagnostic accuracy studies and AI, (2) evidence and statements suggesting modifications to current items, (3) evidence and statements suggesting additions of items, and (4) evidence and statements suggesting the removal of specific items.

#### Meta-Research Study

As previously noted, there have been no studies examining the adherence and suitability of QUADAS-2 for the appraisal of AI diagnostic accuracy study quality. Therefore, a meta-research study was carried out to evaluate the adherence of AI diagnostic accuracy systematic reviews to the existing QUADAS-2 tool. This study demonstrated that there is incomplete uptake of quality assessment tools, as well as inconsistent reporting of bias in AI-diagnostic accuracy systematic reviews, with just over half of the studies using QUADAS-2. This study also identified key biases and features unique to AI diagnostic accuracy studies. These will contribute to the formulation of candidate items for addition or modification.

#### Online Scoping Survey

The project team and steering committee will undertake a survey of an international panel of experts in order to identify potential further items or modifications that warrant inclusion in QUADAS-AI. A diverse and independent panel of experts will be identified by the Project Team and Steering Committee from the various stakeholder groups outlined above. They will be provided with an information sheet describing the study and asked to participate in an online questionnaire. Participants will be asked to consider whether each item on the existing QUADAS-2 tool should be retained, removed, or modified in the QUADAS-AI tool. Free-text sections will allow participants to express their thoughts on each item as well as suggest modifications or further considerations. Furthermore, participants will be asked to comment on additional candidate items or considerations produced from preceding rounds of the item generation process.

#### PPIE Exercise

Finally, a focus group will be conducted with patients and members of the public who have expressed an interest in participating in forums related to digital health and AI. The objective of these discussions is two-fold: (1) to further identify issues not uncovered during previous evidence generation steps and (2) to gain further understanding of the perceived importance to the public of specific items that have been raised thus far. These discussions will be conducted remotely using Zoom (Zoom Video Communications).

An expert facilitator will lead a discussion on the current uses of AI in health care, including considerations on the aims of QUADAS-AI and the important items that the participants deem to be important to capture during the study process. As stakeholder discussions will be conducted virtually on Zoom, anonymized post hoc discussion transcripts will be retained.

#### Collation of Items

The project team and steering committee will group items from the item generation phase into domains and subsequently word items as signaling questions. An online discussion among the project team and members of the steering committee will be held to further refine the domains and signaling questions into a draft tool, which will then enter the Delphi consensus process for approval and refinement.

#### Modified Delphi Consensus Process

We will adopt a pragmatic modified Delphi consensus methodology. The Delphi consensus methodology is a well-established method of obtaining a collective opinion from a group of experts through a series of questionnaires; each one refined based on feedback from respondents on a previous version [15]. We will conduct the Delphi consensus process in a similar way as described in the STARD-AI protocol [10].

Participants from across the world are invited to join the QUADAS-AI Consensus Group on account of their expertise as clinician-scientists, computer scientists, journal editors, EQUATOR Network representatives, epidemiologists, statisticians, health technology industry leaders, funders, health policy makers, legal experts, and bioethicists. The steering committee will identify potential participants from their wider professional network or experts who have made significant contributions to their respective fields. Invited experts will be provided with a written invite detailing the study and given a 6-week timeframe to respond. Those who accept the invitation will be invited to complete each round of the modified Delphi consensus process and will be acknowledged as an author, within a group authorship model, in the publication that arises from this study. Studies of similar scope and breadth, such as STARD-AI, recruited over 150 participants from varied backgrounds across the world. A similar number is anticipated for QUADAS-AI.

During each phase of the modified Delphi consensus process, participants will use a 5-point Likert-like scale to evaluate each item (1—very important, 2—important, 3—moderately important, 4—slightly important, and 5—not at all important). The threshold for consensus will be predefined at ≥75%. Items that achieve ≥75% ratings of 1 or 2 will be put forward for discussion in the final round, which will occur in the form of an online teleconference meeting. Items that achieve ≥75% ratings of 4 or 5 will be excluded. Items that do not meet the 75% consensus threshold will advance to the next phase of the Delphi process. Participants will also have the opportunity to propose additional items that they believe warrant discussion in future rounds through open-ended responses.

In subsequent rounds, the survey will be composed of items for which consensus was not achieved and any new items suggested in prior rounds. Each item will be accompanied by a reminder of the participant’s last rating and the average rating from all participants in the prior round. This allows participants to reconsider their initial evaluations with the benefit of understanding the perspective of the wider group. Items that have not reached a consensus will be put forward for discussion in the following rounds until a consensus is reached. We will conduct descriptive statistical tests on the results for each round (median, range, mean, percentage agreement, and consensus).

Once a consensus is reached, there will be a final meeting between a small group of the project team and the steering committee to finalize the structure and content of the QUADAS-AI tool based on feedback from the Delphi consensus. The primary objective is to develop a draft version of the QUADAS-AI tool. As recommended in the Core Outcome Measures in Effective Trials (COMET) handbook, the nominal group technique, a highly structured group interaction framework, will be used to aid this process [16,17]. Following a brief introduction and explanation of the purpose of the meeting by the facilitators, participants will discuss the inclusion and exclusion of candidate items and share any comments until all contributions are exhausted. This discussion phase will be led by the facilitators to ensure that the discussion will not be dominated by any one individual and be as neutral as possible [18].

The first 2 rounds of the modified Delphi consensus process will be conducted as online surveys using the Delphi Manager software (version 4.0; Embarcadero Technologies), which is developed and maintained by the COMET initiative. The final meeting to draft the QUADAS-AI tool will be conducted using Zoom. All data are pseudo-anonymized and no identifiable data will be published.

#### Development of the Quality Assessment Tool, Statement, and Explanation and Elaboration Document

Upon completion, the project team will construct the initial QUADAS-AI tool. The draft tool, with an accompanying statement, will be shared among the wider steering committee in order to discuss its content and, therefore, allow the steering committee to suggest additions, subtractions, or modifications as they see fit.

#### Piloting Among Experts and Nonexperts

Upon completion of the first draft of the QUADAS-AI tool, we intend to organize multiple rounds of piloting among expert and nonexpert users (QUADAS-AI Pilot Group). The main aim of these piloting sessions is to test the tool’s usability, as well as identify items that are considered to be vague, ambiguous, or perceived to be missing. We intend to undertake this process among health care professionals, computer scientists, expert statisticians, journal editorial boards, key industry stakeholders, regulatory leaders, as well as policy experts. Interviews among this QUADAS-AI Pilot Group will be undertaken in order to ensure that a granular level of feedback is attained for points of discussion. Members of the pilot group will not be part of the steering committee or have previously participated in the consensus process in order to provide an independent opinion. We anticipate around 20 to 30 members will be recruited. Experts and nonexperts within the Pilot Group will be acknowledged by name as author, within a group authorship model, in the publications that arise from this study.

In conjunction with this piloting process, the project team will prepare the explanation and elaboration document, to provide rationale for the domains, structure, and items associated with the tool.

### Stage 3: Dissemination

#### Overview

Following the piloting phase, the final proposed amendments to QUADAS-AI will be discussed among the project team and the steering committee. Once consensus has been reached through email correspondence, the documents will be disseminated.

We strongly anticipate that the dissemination strategy will be principally tailored toward five groups of stakeholders: (1) academia, (2) policy, (3) guidelines and regulation, (4) industry, and (5) patient-representative bodies. Although a significant amount of material will cross over between stakeholders, creating stakeholder-specific material is considered to be the most meaningful way of achieving impact.

#### Academic Stakeholders

We aim to publish the QUADAS-AI tool, the accompanying statement and the explanation and elaboration document in an open-access format in a high-impact, peer-reviewed journal. In order to further complement this, we aim to create specialty-specific discourse regarding QUADAS-AI through focused editorials in pertinent journals. These journal editors will also be actively encouraged to endorse the use of QUADAS-AI as part of their peer review process. Translations of the tool in various languages are also encouraged in order to further broaden the scope of its impact. We urge interested parties to contact the corresponding author for further information about the translation policies.

#### Policy Stakeholders

We are in close collaboration with organizations such as Public Health England, National Health Service (NHS) Digital, National Institute for Health and Care Excellence (NICE), and the NHS Accelerated Access Collaborative (AAC) and their wider network to ensure that the tool will form part of their health technology assessment pathways.

#### Guidelines and Regulatory Stakeholders

QUADAS-AI has been co-designed with senior figures from the United States Food and Drug Administration (FDA) and the United Kingdom Medicines and Healthcare products Regulatory Agency (MHRA). While they do not represent the views of either organization, these steering committee members have a high-level understanding of how QUADAS-AI may be constructed to achieve maximal real-world impact.

#### Industry Stakeholders

We will present QUADAS-AI to a broad range of health technology companies, ranging from start-ups, small, and medium-sized enterprises to multinational corporations, so that their product pipelines may accommodate this.

#### Public and Nonspecific Stakeholders

Ensuring that the core material is available in an open-access fashion, through a CC-BY license, is paramount to achieving general impact. In addition, we aim to publish papers in mainstream media and attain distribution through nontraditional means (eg, social networking platforms, webinars, podcast episodes, and blog posts).

### Ethical Considerations

Ethics approval for the study has been granted by the Joint Research Compliance Office at Imperial College London (21IC6664). Written consent will be gained for all participants in the online scoping survey, PPIE, Delphi consensus process, and checklist piloting.

## Results

As of July 2024, the project team and steering committee have been established, as has the scope of the project. The study is currently in the item generation phase (stage 2), and the mapping and meta-research reviews have been completed. We aim to conduct the scoping survey of experts, PPIE, and Delphi consensus process by the end of 2024 and publish the statement by the first quarter of 2025 for stakeholder use.

## Discussion

QUADAS-AI will be a consensus-derived quality assessment tool that will allow readers to critically appraise the risk of bias and the applicability of study findings in systematic reviews of diagnostic accuracy studies using AI. By providing a framework to evaluate the methodological quality of studies, stakeholders will be in a better position to assess the evidence base and potential for clinical translation of AI-driven diagnostic tools.

AI technology will likely be integrated into several clinical workflows within the next decade in order to enhance patient care and improve clinical outcomes. Specifically, clinical diagnostics has emerged as a key area that has gathered significant interest from global clinical, academic, and industry communities. The importance of evidence synthesis becomes increasingly evident as rapidly advancing AI technology continues to be applied within the diagnostic field; this is typically achieved with systematic reviews to draw clinically relevant conclusions from summarized findings. Therefore, robust methods to evaluate evidence synthesis will be fundamental to the clinical development and implementation of AI technologies as the research community continues to harness the unique ability of AI to generate and process ever-increasing amounts of health data. However, given the notable flaws in using current quality assessment tools, there is a pressing need to develop an AI-specific quality assessment tool that can suitably assess the unique nature of AI diagnostic accuracy studies. We hope that this international, multistakeholder consensus approach will sufficiently address the unique considerations of AI technology, and will ultimately provide a useful tool for clinical, academic, policy, regulatory, and industry stakeholders.

## Abbreviations

AAC: Accelerated Access Collaborative
AI: artificial intelligence
COMET: Core Outcome Measures in Effective Trials
EQUATOR: Enhancing the Quality and Transparency of Health Research
FDA: Food and Drug Administration
MHRA: Medicines and Healthcare products Regulatory Agency
NHS: National Health Service
NICE: National Institute for Health and Care Excellence
PPIE: patient and public involvement and engagement
PROBAST: prediction model risk of bias assessment tool
QUADAS: quality assessment of diagnostic accuracy studies
QUADAS-AI: artificial intelligence–specific quality assessment of diagnostic accuracy studies
ROBIS: risk of bias in systematic reviews
STARD-AI: Standards for Reporting Diagnostic accuracy studies

## Data Availability

Datasets generated or analyzed during this study are available on reasonable request from the corresponding author.
